# Robust Thermochromic Photothermal Coating with Ultraslippery Anti-icing/Deicing and All-Season Temperature Regulation Performance

**DOI:** 10.34133/research.1285

**Published:** 2026-06-01

**Authors:** Shize Sun, Xiaolin Liu, Zelinlan Wang, Changjun Yang, Jichen Chen, Zehui Zhao, Junbo Liu, Liwen Zhang, Huawei Chen

**Affiliations:** ^1^ Institute of Bionic and Micro-Nano Systems, School of Mechanical Engineering and Automation, Beihang University, Beijing 100191, China.; ^2^ State Key Laboratory of Bioinspired Interfacial Materials Science, Bioinspired Science Innovation Center, Hangzhou International Innovation Institute, Beihang University, Hangzhou 311115, China.; ^3^ College of Mechanical and Transportation Engineering, China University of Petroleum, Beijing 102249, China.

## Abstract

Photothermal materials with high conversion efficiency offer a promising approach for preventing ice accretion on infrastructures like wind turbines. However, the conflict between weak-light inefficiency in winter and overheating hazards in summer remains a challenge for composites. Herein, we develop a robust thermochromic photothermal icephobic coating with switchable solar-driven anti-/deicing and anti-overheating modes for all-season demands. During freezing winter, a 0.05 W/cm^2^ weak solar irradiation can raise the black coating (solar absorbance >97%) temperature from −10 to 7.6 °C and facilitate rapid ice shedding from rotating rotor. During summer, the thermochromic coating turns white with strong reflection and limits coating temperature below 51 °C, inhibiting composites oxidation above 60 °C. Strikingly, the interpenetrating elasticity and ultraslippage endow the coating with exceptional ice detachment properties, exhibiting an ultralow ice adhesion strength (<31 kPa) and sliding angle (<8.3°), which are maintained even after 200 Taber abrasion cycles. This study successfully addresses the critical challenge of regulating the all-season temperature of photothermal coatings, pioneering a new pathway for designing intelligent anti-icing coatings for wind turbines and low-altitude rotorcraft.

## Introduction

Ice accumulation poses a critical risk to the structural integrity and operational safety of critical infrastructure, including wind turbine blades, low-altitude rotorcraft, and aerospace systems [1–4]. Various passive and active anti-icing strategies have been developed to mitigate these risks [5–9], depending on whether there is an external energy input. Passive methods typically utilize energy-free functional surfaces, such as superhydrophobic surfaces [10–13], slippery liquid-infused porous surfaces [14–18], and modulus mismatch-driven ice detachment surface [19,20], inhibiting ice accretion and reduce ice adhesion via air/lubricant films interfacial isolation. However, the poor mechanical durability of micro-nano structures and inevitable depletion of air/lubricant films pose fundamental barriers to their application on wind turbines [21]. Active strategies utilize external mechanical, electrical, or solar energy to achieve efficient ice inhibition or removal via force or heat, offering a typically reliable solution [22]. Among them, electrothermal materials offer a direct and efficient anti-icing alternative owing to the easy availability of electric energy, but their large-scale application on wind turbine blades is also prohibited due to potential risks of lightning strikes as well as the extensive electricity consumption.

Photothermal materials offer a very promising method for preventing ice formation on wind turbine blades due to the low cost and renewability of solar energy [23–27]. Continuous attention has been paid to enhance the photothermal conversion efficiency [28–30], by introducing light-absorbing materials (carbon, nanoparticle, semiconductor) and optimizing the light trapping structure (honeycomb, compound-eye, and butterfly wing scales), and enabled temperature rise to 100 °C within a short period of time [31,32]. However, their all-season applications are still restricted by the conflict between weak-light inefficiency in winter and overheating hazards in summer: (a) The inherent intermittency of solar irradiation, including weak light during icing and nighttime absence, markedly reduces deicing efficiency [21,33], and (b) the high temperature and strong solar irradiation during summer will amplify the photothermal effect and pose overheating hazards [34,35], inducing irreversible oxidation or thermal decomposition in the composite substrates of wind turbine blades [36–38]. Developing an all-season adaptable strategy with both high anti-icing/deicing efficiency under weak-light conditions in winter and temperature-limit function in summer becomes an urgent task.

Herein, we report a robust anti-/ deicing coating with temperature-self-adaptive photothermal modes switching, elastic fracture promotion, and ultraslippage characteristics and successfully overcome the critical challenge of photothermal coating with seasonal switching demands. The temperature self-adaptability is achieved via thermochromic microcapsules (TCMs) capable of autonomously shifting between photothermal heating and temperature limit modes in response to ambient temperature. We demonstrate that the coating achieves rapid and large-area ice detachment under low-temperature conditions, along with resisting natural mechanical damage caused by abrasion objects, attributed to the interpenetrating of rigid E51 epoxy resin (E51-EP) chains with flexible α, ω-polysiloxane organogel. This coating is systematically evaluated in real-world application scenarios, including ice adhesion reduction, anti-icing, and deicing, under weak solar radiation. Furthermore, we demonstrate the dynamic deicing capability of coated wind turbine blades, highlighting their potential for practical applications. This work not only provides a scalable manufacturing route for intelligent dual-mode anti-icing surfaces but also offers new insights into the design of next-generation adaptive coatings with enhanced durability and environmental versatility.

## Results and Discussion

### Design, fabrication, and morphologies of TA-SIDI coatings

**Fig. 1.**
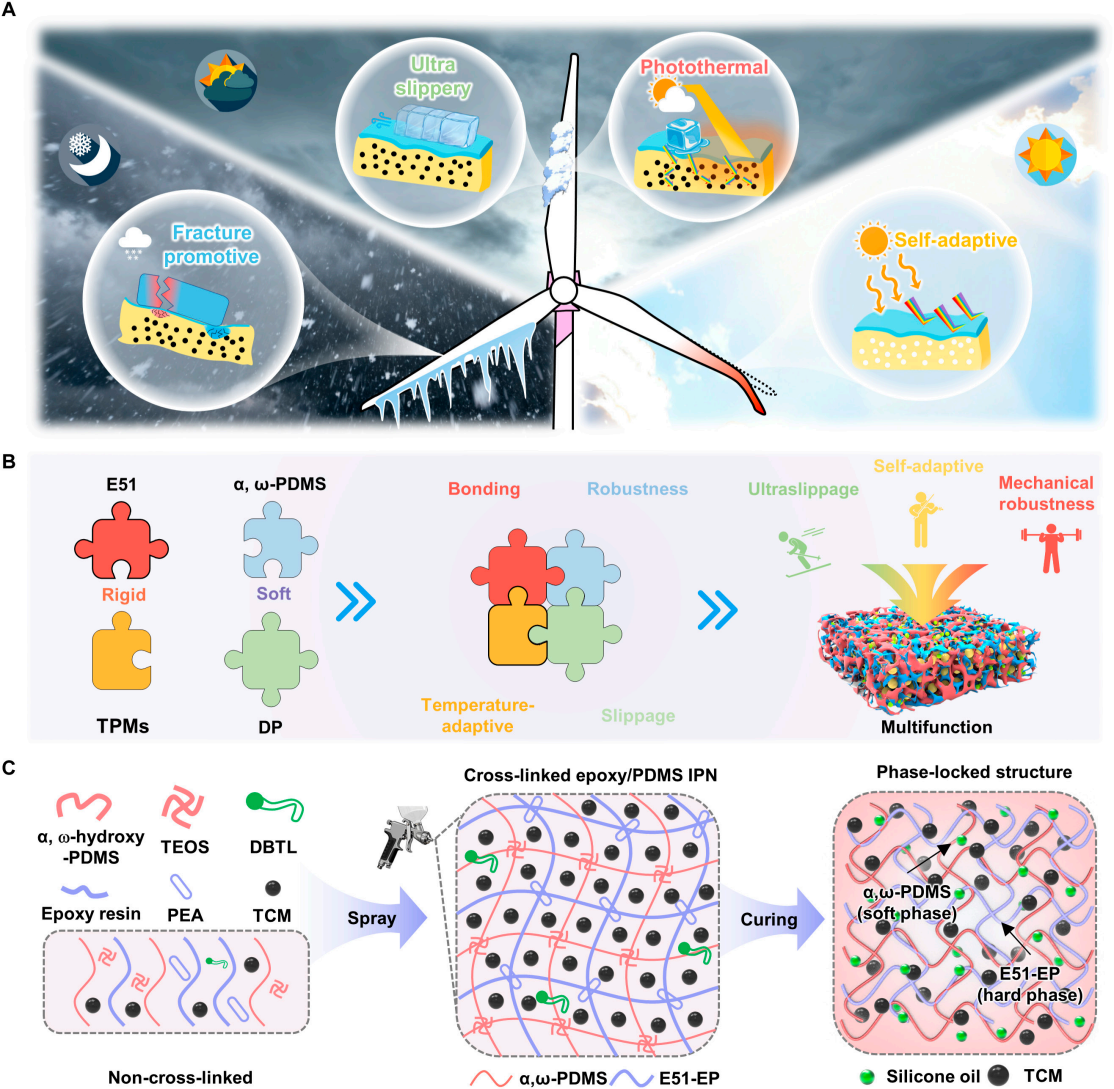
Design, interpenetrating synthesis strategy, and synthesis method of temperature-self-adaptive ultraslippery ice detachment interface (TA-SIDI). (A) Wind turbine blades face a dilemma: weak-light anti-icing/deicing inefficiency in winter and overheating hazards in summer. Corresponding strategy: all-season self-adaptive photothermal coating with ultralow ice adhesion, excellent mechanical robustness, and thermochromic ability. (B) Schematics of the multiscale molecular interpenetrating coupling structure and composite performance enhancement mechanism. (C) Synthesis process of TA-SIDI.

Anti-icing coatings must fully use all available energy to overcome the challenge in various extreme environments. The photothermal coating with high energy conversion efficiency successfully delays ice formation on the surface and substantially promotes ice removal [33,39]. Meanwhile, to prevent the photothermal coating from overheating, the coating must be capable of temperature limit to adapt to hot summer environments (Fig. 1A).

Through the strategic fusion of TCMs and interpenetrating polymer network (IPN) within a multiscale hierarchical architecture, temperature-self-adaptive ultraslippery ice detachment interface (TA-SIDI) achieves synergistic integration of diverse material properties, optimizing the inherent advantages of individual components while enabling unprecedented multifunctionality [40]. Therefore, TA-SIDI not only exhibits the capability of temperature-adaptive color switch but also demonstrates the superior mechanical strength of E51-EP and strong bonding with the substrate. Additionally, it possesses the organic gel characteristics and lipophilicity of α, ω-dihydroxyl polydimethylsiloxane (α, ω-PDMS). α, ω-PDMS and liquid silicone oil are highly similar in chemical composition. Liquid silicone oil can be fully impregnated as a dispersed phase (DP) within the α, ω-PDMS network to form a uniform mixed system, and it can further maintain a stable state through intermolecular forces (Fig. 1B).

Figure 1C illustrates the synthesis process of TA-SIDI. We introduced E51-EP chains into the α, ω-PDMS organogel to acquire the IPN. The mechanical robustness and performance of the anti-icing coating can be substantially improved by utilizing the synergy of a high mechanical strength skeleton and low surface energy. The temperature-self-adaptive ice-detachment interface (TA-IDI) was prepared by mixing the TCMs with the IPN. At this point, the interface can achieve a relatively low ice adhesion strength and realize the photothermal effect by absorbing visible light via TCMs. After the coating is completely cured, the liquid lubricant silicone oil is permeated and adsorbed into the voids of α, ω-PDMS through diffusion, and TA-SIDI is obtained with ultraslippage performance, markedly minimizing the stress threshold of fracture initiation for ice detachment. The main difference between TA-IDI and TA-SIDI is the existence of a liquid lubricant.

### Temperature-regulating optical properties, wettability, and complementary performances of TA-SIDI

**Fig. 2.**
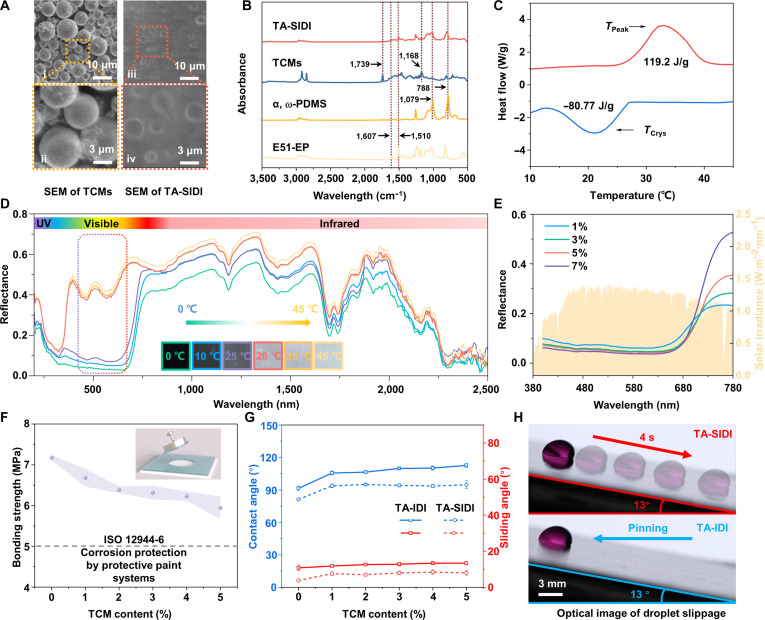
Characterization, optical properties, wettability, and complementary performances of temperature-self-adaptive ultraslippery ice detachment interface (TA-SIDI). (A) Scanning electron microscopy (SEM) image of the thermochromic microcapsules (TCMs) and TA-SIDI coating. (B) Fourier transform infrared (FT-IR) spectra of TA-SIDI, TCMs, α, ω-Dihydroxyl polydimethylsiloxane (α, ω-PDMS), and E51-EP. (C) Heating and cooling thermograms of TCMs by differential scanning calorimetry (DSC). (D) Solar reflectance spectrum of TA-SIDI coating varying with temperature. The inset depicts the color response of the TA-SIDI coating at different temperatures. (E) Solar reflectance spectrum of TA-SIDI coating varying with TCM content in the visible spectrum. (F) Bonding strength of TA-SIDI as a function of the TCM content. The TA-SIDI coating complies with the requirements of ISO (International Organization for Standardization) 12944-6 “Corrosion Protection of Protective Paint Systems”. (G) Contact angle and sliding angle of TA-SIDI and temperature-self-adaptive ice-detachment interface (TA-IDI) with different TCM content. (H) Optical image of droplet slippage on TA-SIDI and TA-IDI. (Note that TA-SIDI always possesses 5% TCMs when there are no special instructions in this work.)

The scanning electron microscopy (SEM) images in Fig. 2A i to ii show that the TCMs are regular spheres with a smooth and nonporous surface, whose average size is about 3 μm (Fig. S1). Figure 2A iii and iv shows that TCMs are uniformly distributed in the IPN. The Fourier transform infrared (FT-IR) spectra of E51-EP, α, ω-PDMS, TCMs, and TA-SIDI are presented in Fig. 2B (Fig. S4). The α, ω-PDMS organogel skeleton was cured by a condensation reaction between tetraethyl orthosilicate (TEOS) and the terminal hydroxyl group of α, ω-PDMS, catalyzed by dibutyltin dilaurate (Fig. S2) [41]. The primary amine groups of poly (propylene glycol) bis (2-aminopropyl ether) (PEA) participated in ring-opening reaction with E51-EP to form a cross-linked rigid skeleton (Fig. S3). There were 2 characteristic peaks of E51-EP at the wave numbers of 1,607 and 1,510 cm^−1^, corresponding to the C–H vibration of the benzene ring of the E51-EP skeleton, respectively. The TA-SIDI and α, ω-PDMS peaks at 788 and 1,079 cm^−1^, respectively, derived from the stretching vibration of Si−O [42]. Further, the absorption peaks at 1,739 and 1,168 cm^−1^ correspond to the specific –COO– groups of TCMs and TA-SIDI. These peaks are typical stretching vibration peaks of E51-EP, α, ω-PDMS, and TCMs, which successfully prove the preparation of the IPN system. Figure 2C shows the prominent melting peak (*T*_Peak_ = 32.79 °C) and cooling peak (*T*_Crys_ = 21.11 °C) of TCMs and the thermochromic temperatures of TCMs. FT-IR and differential scanning calorimetry curves fully clarify the composition of TA-SIDI and indicate the successful preparation of the multiscale molecular interpenetrating structure.

With a temperature range from 0 to 45 °C, the TA-SIDI coating exhibits a color shift from black to white (Fig. 2D and Fig. S5). In particular, within the temperature range of 25 to 28 °C, the color of the coating undergoes a sudden change, correspondingly modulating its maximum visible reflectance (from 380 to 780 nm, visible spectrum; Fig. 2D) from ~3% to ~57%. This feature enables TA-SIDI to adaptively adjust the photothermal conversion efficiency according to the ambient temperature, thereby achieving intelligent switching between photothermal heating mode and temperature limit mode. The uniform distribution of TCMs within the coating endows the coating with excellent light absorption capacity below 28 °C. With the increase in the concentration of TCMs in the coating, the ability to achieve light reflection at room temperature gradually decreases. Adding only 1%wt of TCMs can achieve a minimum reflectivity of 7%. TA-SIDI, containing 3% to 5%wt of TCMs, exhibits similar light reflection properties, and the lowest light reflectivity is respectively 4.6% and 4.2%. When the concentration of TCMs reaches 7%, the light reflection ability can be as low as 3.7% (Fig. 2E). Considering the influence of TCM content on coating strength and light reflectivity, TA-SIDI always contains 5%wt TCMs, unless otherwise specified in this work.

The coating further enhances bonding strength and mechanical robustness by employing the interpenetrating network strategy. Due to the mutually independent curing reactions, the interpenetration of α, ω-PDMS and E51-EP can occur in any proportion. Referring to our previous work [11], a 1:1 IPN ratio was selected. Figure 2F depicts the relationship between the TCM concentration and the bonding strength of the coating. The substrate bonding strength of the TA-SIDI coating with 5%wt TCMs can still reach ~5.9MPa, which is only 18% lower than that of pure E51-EP (Fig. S6). The bonding strength of the TA-SIDI coating exceeds the 18.9% requirement set by ISO (International Organization for Standardization) 12944-6 (the bonding strength of the protective paint systems for corrosion protection should be higher than 5 MPa.). Figure 2G shows the contact angle and sliding characteristics of the TA-SIDI coating as a function of the TCM content. The contact angle of the coating without TCMs is merely 91°. When the mass ratio of TCMs reaches 5%, the contact angle of TA-IDI reaches 112° (Fig. S7), and the sliding angle is 13.4°. The characteristics of the organogel and lipophilicity of TA-IDI enable DP to be fully impregnated and form a continuous lubricating effect on the surface [43]. This further reduces the sliding angle of the coating to less than 10°. TA-SIDI demonstrates excellent sliding behavior (Fig. 2H), markedly reducing the residence time of droplets on the surface.

### The photothermal and temperature limit properties of TA-SIDI

**Fig. 3.**
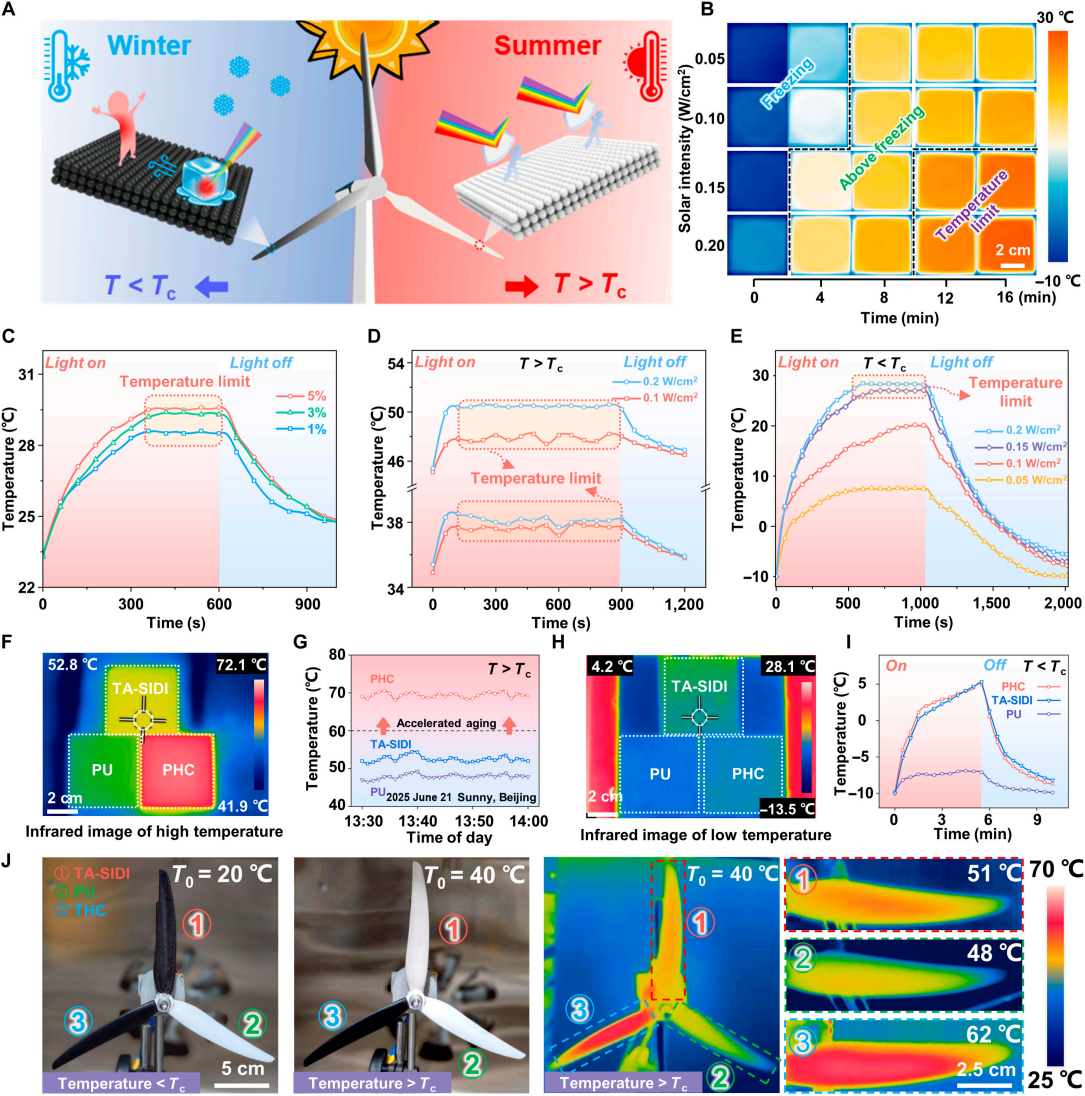
Photothermal heating and temperature limit performance of temperature-self-adaptive ultraslippery ice detachment interface (TA-SIDI). (A) Schematic of temperature-regulating self-adaptive coating. (B) Thermal infrared images of TA-SIDI with different solar intensities when the environmental temperature is −10 °C. (C) Photothermal process curves of TA-SIDI with different thermochromic microcapsule (TCM) content, where the plateaus in the temperature curves indicate the temperature exceeds *T*_c_ and transitions to the limit state. (D) Photothermal process curves of TA-SIDI with 0.1 and 0.2 W/cm^2^ solar intensity with a hot plate. (E) Photothermal process curves of TA-SIDI with 0.05, 0.1, 0.15, and 0.2 W/cm^2^ solar intensity in a low-temperature environment. (F) Thermal infrared images of samples covered with TA-SIDI, PU (polyurethane), and PHC (photothermal heating coating) coatings at a temperature of ≈35 °C. (G) The real-time temperature record of the TA-SIDI, PHC, and PU coating (Beijing, 2025 June 21, sunny). (H) Thermal infrared images of samples covered with TA-SIDI, PU, and PHC coatings at a temperature of ≈−10 °C. (I) Photothermal process curves of TA-SIDI, PU, and PHC coating under 0.05 W/cm^2^ solar intensity in a low-temperature environment. (J) Temperature limit performance of TA-SIDI under simulated high-temperature environment (*T*_0_ is the temperature of the environment).

Figure 3A illustrates the working mechanism of the TA-SIDI coating. When the temperature falls below the critical temperature (*T*_c_, ~28 °C for TA-SIDI coating), TA-SIDI exhibits a black appearance, substantially enhancing its visible light absorption capacity. Through the photothermal effect, this enhanced absorption elevates the surface temperature, effectively suppressing surface ice formation. When the TA-SIDI coating rises to the critical temperature due to the spontaneous photothermal effect or the ambient temperature, the coating undergoes a phase transition and becomes white. This color modulation activates the temperature limit state, thereby suppressing further temperature elevation and achieving intelligent management of the coating temperature. The infrared thermal images during the temperature rising process of the sample are shown in Fig. 3B (inset), manifesting that the TA-SIDI has excellent photothermal and temperature limit capabilities. Even at an extremely low solar intensity of 0.05 W/cm^2^, the coating temperature rose from −10 to 7.4 °C after 600 s, demonstrating excellent anti-icing performance. When the solar intensity increased to 0.2 W/cm^2^, the coating rapidly rose to 28 °C and then switched to the temperature limit state, suppressing further increases in coating temperature. The coating temperature then stabilized at 28.4 °C. Figure 3C shows the photothermal effect of TA-SIDI with different mass ratios of TCMs, ranging from 1%wt to 5%wt. The samples were irradiated for 600 s using a solar simulator, and the average temperature curve of TA-SIDI was recorded. Upon solar irradiation, the TA-SIDI coating with a mass fraction of 5%wt exhibits excellent photothermal conversion ability, elevating its temperature to *T*_c_. Subsequent gradual thermal equilibration stabilizes the surface temperature at 29.5 °C, demonstrating its intelligent thermal regulation. When the proportion of TCMs in the TA-SIDI decreases from 5% to 1%, the equilibrium temperature of the sample surface drops to 28.6 °C. This indicates that even at extremely low concentrations of TCMs, the TA-SIDI coating can still exhibit a good photothermal heating effect. Figure 3D shows the unique temperature limit ability of TA-SIDI at *T* > *T*_c_. We recorded the temperature of the TA-SIDI coating surface under different solar intensities at ambient temperatures of 35 and 45 °C. Under an environment with a solar intensity of 0.2 W/cm^2^ and a temperature of 35 °C, the surface temperature of the TA-SIDI coating reached a maximum of 38.6 °C at 120 s and remained stable at ~38 °C. It is worth noting that under a solar intensity of 0.2 W/cm^2^, the coating in an ambient temperature of 45 °C only rose to 50.6 °C to reach equilibrium. Compared with the existing highly efficient photothermal strategies [29,31,32], the temperature has been markedly reduced by approximately 40 °C (Fig. S8). The data indicate that when the environmental temperature exceeds *T*_c_, even under intense solar intensity of 0.2 W/cm^2^, the temperature of the TA-SIDI will only rise slightly, demonstrating excellent temperature limit capability. The photothermal effect under the freezing point is investigated by a low-temperature chamber (Fig. 3E, the temperature variation of TA-SIDI during the cyclic heating–cooling process at 0.20 W/cm^2^ solar intensity, as shown in Fig. S9). The samples were exposed to an ambient environment at −10 °C with a relative humidity of 40% ± 5%. The photothermal performance varies notably with the solar intensity. These results indicate that the TA-SIDI coating has the ability to switch between 2 modes of photothermal heating and temperature limit, effectively mitigating the icing problems caused by the deployment of traditional radiative cooling materials in cold weather, while avoiding overheating caused by conventional photothermal coatings in hot weather.

The temperature limit function of the TA-SIDI coating is particularly effective in outdoor scenarios, especially when used as a protective coating for wind turbine blades, resulting in substantial energy savings. The performance of TA-SIDI is evaluated by simultaneously placing TA-SIDI, PHC (photothermal heating coating), and PU (polyurethane) in the same environment. Among them, PHC is a conventional noncolor-change, single-mode photothermal material based on carbon nanotubes, and PU is a polyurethane exterior paint for wind turbines. At a higher temperature (2025 June 21, Beijing, sunny, *T* > *T*_c_), the infrared images of Fig. 3F revealed that the surface temperatures of both TA-SIDI and PU were notably below that of the PHC coating. In addition, judging from the digital photos, the TA-SIDI coating exhibited a white appearance similar to that of the PU (Fig. S10). The highest temperature on the infrared image shows a pronounced contrast. The ambient temperature is about 35 °C. Under a solar intensity of 0.1 W/cm^2^, the PHC coating continuously heated up and reached ~70 °C. However, the coating temperature of TA-SIDI was only 54.3 °C, which was 15.7 °C lower than that of the PHC coating and only 4.1 °C higher than that of the PU coating (Fig. 3G). This indicated that TA-SIDI can provide a cooling effect similar to pure PU. Compared to the PHC coating, it can be found that the traditional single-mode photothermal coating can rise to 70 °C in a short time when exposed to sunlight, which will cause rapid aging and irreversible damage to the composite materials of wind turbine blades [44]. At a lower temperature, it can be observed that the temperatures of the TA-SIDI coating and the PHC coating are higher than those of the PU coating (Fig. 3H and Figs. S11 and S12). This is attributed to the fact that both TA-SIDI coating and PHC coating can absorb visible light through the photothermal effect, causing the surface temperature to rise and exceed the freezing point. Real-time temperature records showed an increase of 15.3 °C relative to the cold plate (Fig. 3I). In sharp contrast, the surface of PU only rose by 3 to −7 °C and is still far below the freezing point, which makes the freezing risk of the PU surface much higher than that of TA-SIDI. To comprehensively evaluate the temperature limit performance of the TA-SIDI coating, simulated wind turbine blades were coated with TA-SIDI (①), PU (②), and PHC (③) coatings for testing (Fig. 3J). When the ambient temperature exceeds *T*_c_, the TA-SIDI coating on the wind turbine blade undergoes a rapid color transition from black to white, leading to a marked reduction in photothermal conversion efficiency. Under 0.1 W/cm^2^ simulated solar irradiation, the infrared images depict that the temperature of blades coated with TA-SIDI was 11 °C lower than that of blades coated with PHC, demonstrating the effective temperature limit capability of TA-SIDI.

The above results indicate that the TA-SIDI coating can adaptively and intelligently switch between the photothermal and temperature limit modes, making it highly suitable for outdoor scenarios requiring year-round temperature management, such as wind turbine blades.

### The mechanical robustness and outdoor durability of TA-SIDI

**Fig. 4.**
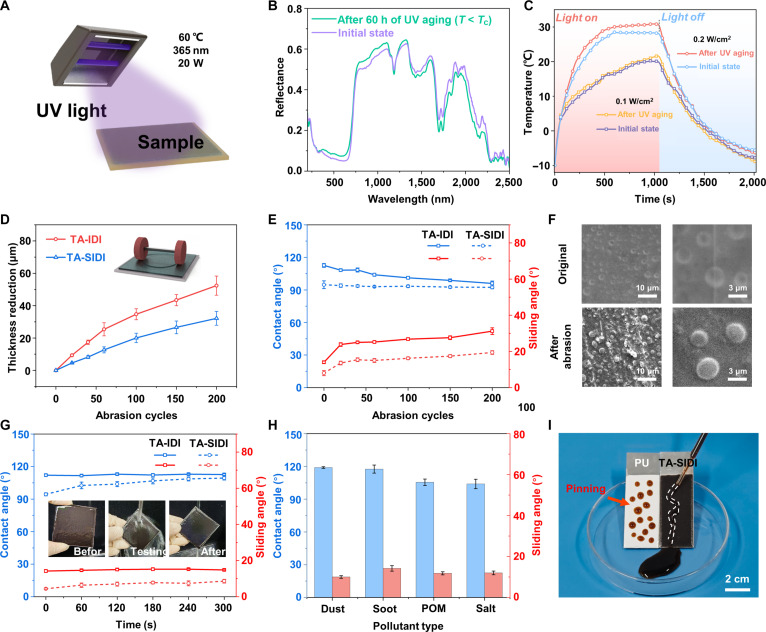
Outdoor durability, mechanical robustness of temperature-self-adaptive ultraslippery ice detachment interface (TA-SIDI) coating. (A) Schematic of the ultraviolet (UV) aging test for TA-SIDI coating. During the UV aging experiment, the environmental temperature was approximately 60 °C, and the wavelength of the UV light was 365 nm with a power of 20 W. (B) The TA-SIDI coating before and after UV accelerated aging test for 60 h at 10 °C (*T* < *T*_c_). (C) Photothermal process curves of TA-SIDI before and after UV accelerated aging test with 0.1 and 0.2 W/cm^2^ solar intensity in a low-temperature environment. (D) Thickness reduction of TA-SIDI and temperature-self-adaptive ice-detachment interface (TA-IDI) after abrasion. (E) Contact angle and sliding angle on the abrasion areas of TA-SIDI and TA-IDI after 200 abrasion cycles. (F) Scanning electron microscopy (SEM) images of TA-SIDI before and after 200 abrasion cycles. (G) Influence of water impact time on the wettability of the TA-SIDI coating. The inset shows the photographs of the high-speed water jet impacting the TA-SIDI coating. (H) Contact angle and sliding angle of different polluting agents, including dust, soot, particulate organic matter (POM), and salt. (I) Photographs of the TA-SIDI (right) and PU (polyurethane) (left) subjected to muddy water.

During the outdoor applications, the material is exposed to environmental factors such as ultraviolet (UV) radiation aging, sand and dust impact, rain erosion, and pollutant accumulation. All these factors may impact the photothermal properties and wettability of the coating. After a 60-h UV aging cycle experiment, the light absorption performance of the coating was tested under *T* < *T*_c_. The TA-SIDI coating can still exhibit optical properties close to the original state (Fig. 4B). By comparing the photothermal heating capacity of the TA-SIDI coating before and after the UV aging experiment (Fig. 4C), it can be found that the coating can still increase by 31.6 °C in 1,020 s under 0.1 W/cm^2^ solar intensity. Under solar intensity of 0.2 W/cm^2^, the temperature of the TA-SIDI coating rose to 30.9 °C after 1,020 s. It has increased slightly by 2.7 °C compared with the initial state, preserving the ability to limit temperature.

Considering that the application of the TA-SIDI coating on wind turbine blades will face extreme wind, snow, and sandstorm weather, the Taber wear resistance test was selected to evaluate the anti-wear performance of the coating. A smaller thickness reduction indicates stronger mechanical robustness of the coating. Taking advantage of the multiscale interpenetrating strategy, the rigid chains of E51-EP reinforce the mechanical strength of the α, ω-PDMS elastomer, and the superficial lubricating effect caused by full impregnation of the skeleton with the DP further reduces the damage after abrasion, which is shown in Fig. S13. After 200 abrasion cycles, the thickness of TA-SIDI decreased by 32 μm. Compared with TA-IDI, it is reduced by nearly 20 μm under the effect of interfacial lubrication (Fig. 4D). Compared with the reported mechanical robust interface (∼160-μm thickness reduction after 100 abrasion cycles under the same load) [45], the synergy between the liquid lubricant effect and the rigid skeleton greatly provided effective countermeasures to prevent TA-SIDI from abrasion and function failure. As a result, after 200 abrasion cycles, the contact angle of TA-IDI decreased from 112° to 96°, and the sliding angle increased from 14° to 31°. Notably, the contact angle of TA-SIDI only decreased by 2.4°, and the sliding angle increased by 11°, demonstrating a robust durability (Fig. 4E). Figure 4F shows the SEM images before and after the abrasion of TA-SIDI. The scratch marks on the coating surface can be seen clearly. The rigid skeleton of E51-EP endows the coating with extremely strong bonding strength, enabling TCMs to remain firmly fixed on the coating surface without peeling off even when the surface layer of TA-SIDI is worn off. The complete interpenetration at different scales endows the TA-SIDI coating with excellent mechanical stability. After a 300-s high-speed jet impact, the TA-IDI coating demonstrated robust performance, as its contact angle and sliding angle remained almost unchanged. α, ω-PDMS is integrated into the skeleton as a lipophilic material. Thus, DP can fully diffuse into the IPN skeleton. The contact angle increased from 94° to 109°, approaching the TA-IDI’s contact angle (~112°, Fig. 4G). The sliding angle only increased from 4.4° to 8.6°, still far lower than the sliding angle of the TA-IDI coating (~14°). The convergence in contact angle suggests that the removal of insufficiently impregnated DP from the surface layer, while the low sliding angle indicates that the lubricity is maintained by the dispersed DP phase within the IPN skeleton rather than by a macroscopic surface oil layer to achieve extremely low friction and ice adhesion. The accelerated contamination tests were further conducted based on the ASTM (American Society for Testing and Materials) D7897-18 standard [46,47]. We recorded the contact angles of different types of pollutants on the coating surface and made comparisons. Various liquids with different surface tension all exhibit sliding behavior on the TA-SIDI. The pollutants on the surface of TA-SIDI present a good wettability. Although the contact angle of particulate organic matter (POM) and salt on the surface performed relatively low, its sliding angle still maintained at 12° (Fig. 4H). Excellent sliding characteristics give TA-SIDI coating a strong antifouling ability. Figure 4I shows that pollutants can be effectively removed by sliding on the surface without leaving residues. On the contrary, due to the lack of antifouling ability of PU, a large amount of pollutant remained on the surface.

### All-season anti-/deicing performance of TA-SIDI

**Fig. 5.**
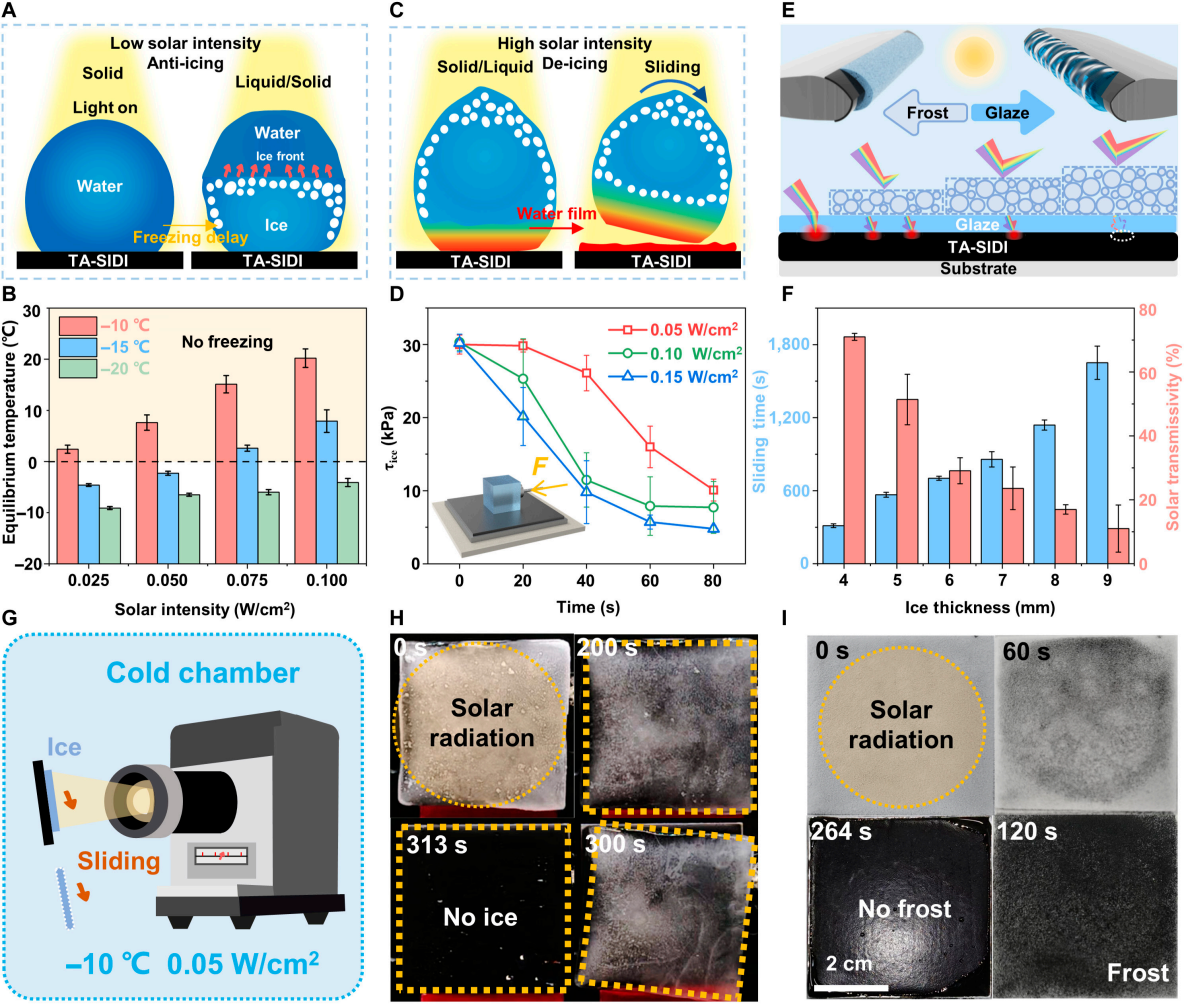
Efficient anti-icing/deicing performance of temperature-self-adaptive ultraslippery ice detachment interface (TA-SIDI) coating. (A) Schematic illustration of the photothermal effect under weak solar intensity for anti-icing. (B) Equilibrium temperature of TA-SIDI with different solar intensities under −10, −15, and −20 °C. (C) Schematic illustration of the photothermal effect under high solar intensity for deicing. (D) *τ*_ice_ of TA-SIDI with different solar intensities as a function of time (Inset: The mechanism of side-push ice debonding. The *τ*_ice_ of the coating was measured at −20 °C with an ice block [1 × 1 × 1 cm^3^]). (E) Schematic of the effect of frost and glaze with different solar transmissivity. (F) Sliding time of the ice on the TA-SIDI surface with different ice thicknesses. (G) Schematic of the ice sliding test for TA-SIDI coating with solar simulator. (H) Ice-sliding performance along with time during the deicing process. (I) Frost-melting performance along with time during the deicing process.

The anti-icing mechanism is explained by analyzing the change at the solid–liquid interface. As depicted in Fig. 5A, the TA-SIDI coating is still capable of attaining a high photothermal conversion efficiency under weak solar conditions. During the initial cooling phase, the droplets remain on the surface of the coating. Subsequently, since the coating can maintain a relatively high temperature and transfer heat to the interior of the droplets, the freezing time is extended. The TA-SIDI coating has demonstrated excellent photothermal conversion performance under various environmental conditions (Fig. 5B and Fig. S14). At a temperature of −10 °C and with a solar intensity of 0.1 W/cm^2^, the TA-SIDI coating can rise to 20.2 °C. Even under an extremely weak solar intensity of 0.025 W/cm^2^, the TA-SIDI coating can still increase by 10.4 °C, exceeding the freezing point and achieving anti-icing. At an ambient temperature of −20 °C and under different solar irradiance conditions, all tested coatings induced a temperature rise of greater than 10 °C, effectively postponing the freezing process of droplets on the surface.

The TA-SIDI coating also shows excellent deicing performance with high solar intensity. Before the deicing process, as shown in Fig. 5C, the TA-SIDI coating is covered with ice. Upon irradiation, the TA-SIDI coating rapidly converts sunlight into thermal energy. The generated heat is transferred upward to melt the underlying ice layer, resulting in the formation of an ultrathin water film at the ice–coating interface. This water film acts as a lubricating layer, enabling the ice droplets to be readily detached upon the application of an external force. The ice adhesion strength (*τ*_ice_), defined as the force (*F*) required to detach an ice sample of a defined contact area (*A*) from a surface, is a standard metric for evaluating surface icephobicity [48,49]. Surfaces with an ice adhesion strength below 100 kPa are generally classified as icephobic [6,48]. The specific side-push debonding method used to measure *τ*_ice_ in this work is illustrated in Fig. 5D (inset). When the ice adhesion strength is reduced to values less than 20 kPa [50], complete melting of the ice is unnecessary. Under such conditions, passive deicing can be accomplished solely by natural forces such as gravity or wind. Figure 5D demonstrates that the TA-SIDI coating possesses a low initial ice adhesion strength of 30 kPa without any photothermal activation. Following exposure to 0.15 W/cm^2^ irradiation for 20 s, the strength precipitously drops to 20 kPa, which is sufficiently low for self-shedding of ice. Further irradiation at a lower intensity of 0.05 W/cm^2^ for 80 s, an ultralow ice adhesion strength of 10.1 kPa was achieved. This successive reduction in ice adhesion may be attributed to the presence of a nanoscale quasi-liquid layer (QLL) on the TA-SIDI surface [51,52]; QLL possesses liquid-like fluidity while its structure lies between that of solid ice and liquid water [53]. Specifically, upon solar irradiation of the TA-SIDI coating, the thickness of the interfacial QLL increases sharply and nonlinearly as the temperature rises toward 0 °C, thereby inducing a rapid decline in ice adhesion strength. Taking advantage of the multiscale interpenetrating strategy, the rigid chains of E51-EP reinforce the mechanical strength of the α,ω-PDMS elastomer, and the surface lubrication effect further mitigates abrasion damage. As shown in Fig. S15, these features ensure that the TA-SIDI coating retains a low ice adhesion strength even after repeated deicing cycles, water impact, and Taber abrasions. The experimental results demonstrated that the TA-SIDI coating exhibits a superior deicing performance under low-light conditions.

The formation of distinct ice types (e.g., clear ice, rime ice, and mixed ice) on wind turbine blades presents a challenge because their optical transmittance differs drastically, thereby constraining the efficacy of photothermal deicing strategies. To overcome this limitation, we explore the coupled photothermal and ultraslippage deicing properties of the TA-SIDI coating under simulated icing conditions (Fig. 5E). As depicted in Fig. 5F, a 4-mm-thick layer of clear ice was formed on the TA-SIDI coating, exhibiting a light transmittance of approximately 71%. At an ambient temperature of −10 °C, the ice was completely detached after 313 s of exposure to solar irradiation at 0.05 W/cm^2^ (Fig. 5G and H). To evaluate the influence of light transmittance on deicing performance, frost layers of varying thickness were deposited atop the 4-mm clear ice. In contrast, when a 5-mm frost layer was deposited on top of the clear ice (total thickness: 9 mm, comprising 4-mm ice and 5-mm frost; transmittance: 11%), a notably longer irradiation time of 1,651 s was required to achieve complete removal. The deicing performance of the TA-SIDI coating under varying frost thicknesses was also investigated (Fig. 5I and Figs. S16 and S17). Complete frost melting was observed within 264 s, demonstrating the coating’s rapid and efficient defrosting capability. These results confirm the exceptional performance of TA-SIDI in mitigating ice and frost accumulation under weak solar conditions of winter.

### Dynamic anti-/deicing performance of TA-SIDI

**Fig. 6.**
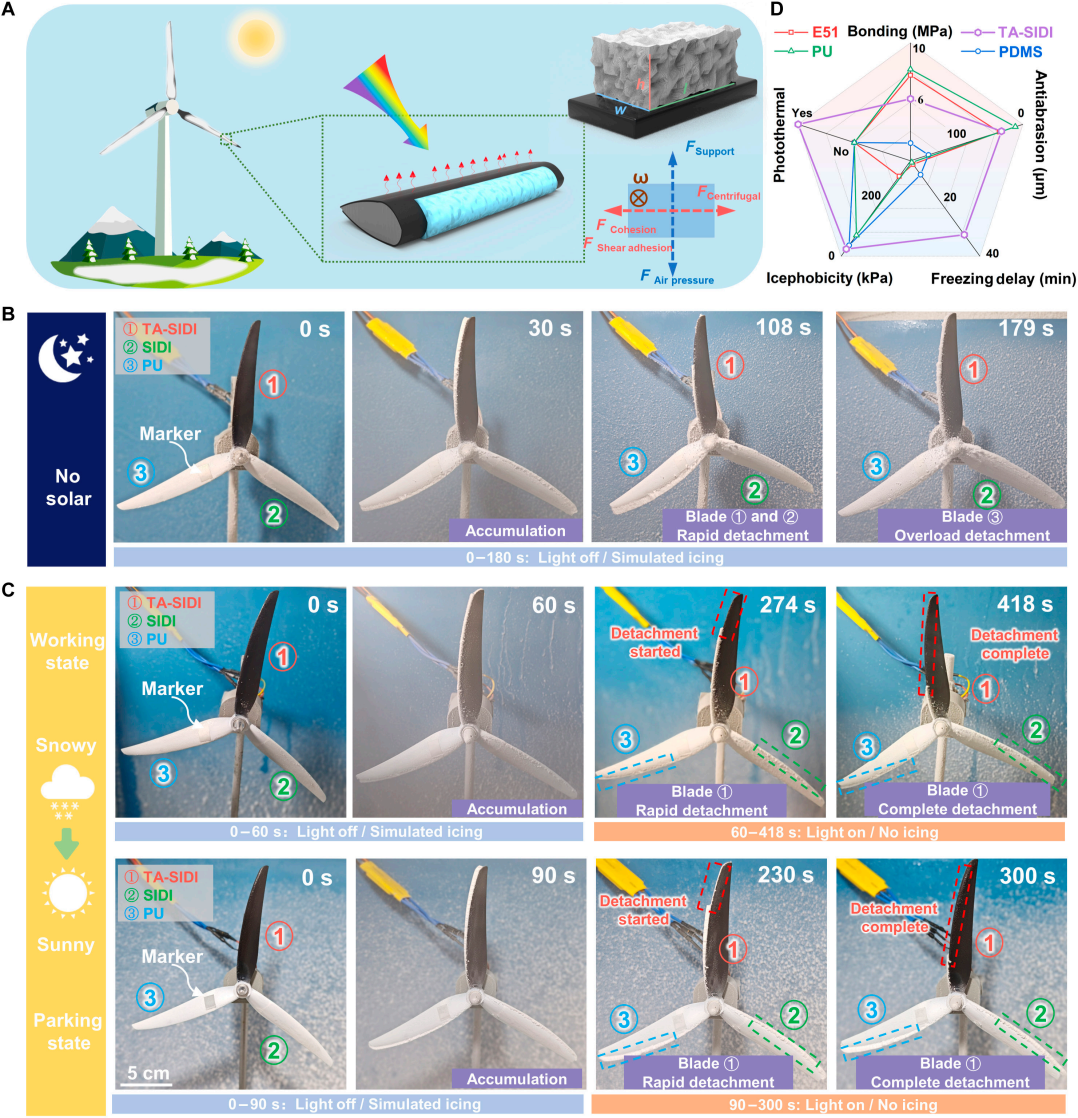
Dynamic anti/deicing performance of temperature-self-adaptive ultraslippery ice detachment interface (TA-SIDI). (A) Schematic of the TA-SIDI with photothermal effect for defrosting on wind turbine blades. The insets show the force analysis at the crack area. (B) Anti-/deicing performance of TA-SIDI during the icing and deicing process with no solar. (C) Anti-/deicing performance of TA-SIDI during the icing and deicing process under solar conditions. Upper panel: Simulation of wind turbine blades with a small amount of snow, in a rotating working state. Lower panel: Simulation of wind turbine blades with heavy snow, in a parking state. (D) Performance distribution graph of TA-SIDI with E51, PU (polyurethane), and PDMS (Sylgard 184).

Ice accreted on wind turbine blades is typically subjected to a combination of centrifugal, cohesive, and shear adhesion forces (Fig. 6A; see the inset for force analysis). Detachment and shedding of ice occur when the centrifugal force generated by blade rotation exceeds the combined cohesive and shear adhesion forces of the ice. An effective deicing coating must therefore maintain its anti-icing performance under dynamic atmospheric conditions, including blizzards and freezing rain. To comprehensively evaluate the dynamic anti-icing performance of the TA-SIDI coating, simulated wind turbine blades were coated with TA-SIDI (①), SIDI (②), and PU (③) coatings for testing. A typical modern 2-MW wind turbine possesses a rotor with a diameter of 90 m, a rotational speed of 9 to 19 rpm, and a tip area’s linear velocity of 23.04 to 89.54 m/s [54]. Therefore, we adopted 1,000 rpm to test the anti-icing ability of TA-SIDI in a dynamic environment. The all-season deicing performance of the TA-SIDI coating, leveraging the synergistic effect of photothermal and ultraslippage, was evaluated under both no-light (Fig. 6B, no solar) and weak-light (Fig. 6C and Fig. S18, 0.05 W/cm^2^) conditions. Experiments were conducted in a low-temperature chamber, whose temperature was maintained at −10 °C. Under no-light conditions, ice accretion led to eventual detachment on all blades. However, the ice detachment time for the TA-SIDI and SIDI coatings (108 s) was markedly shorter than that for the PU control groups (179 s), highlighting their superior low ice-adhesion properties. As shown in Fig. 6C, the transition from snowy to sunny conditions was experimentally simulated. The photothermal ice-removal performance of the wind turbine blades was evaluated under 2 distinct scenarios: under rotating conditions with less snow accumulation (upper panel) and under stationary conditions with heavy snow accumulation (lower panel). To simulate less ice accumulation, the blades were operated for 60 s in an icing environment. After 60 s, simulated solar irradiation was turned on, and partial ice detachment first occurred at the edge of blade ① (TA-SIDI) at 274 s, followed by complete ice shedding at 418 s. In contrast, ice remained adhered to blades ② (SIDI) and ③ (PU) throughout the experiment. For the heavy-ice-accumulation scenario, the blades were first rotated for 90 s in the simulated icing environment. After the solar was activated at 90 s, the blades began rotating after 140 s of irradiation, resulting in ice shedding. Following totally 210 s of irradiation, the ice on blade ① (TA-SIDI) completely detached, whereas the ice on blade ② (SIDI) remained attached due to the lack of a photothermal effect. These results demonstrate that the photothermal effect markedly reduces the ice detachment time, thereby effectively mitigating ice-accretion-induced damage to wind turbine blades.

The synergistic integration of photothermal conversion and ultraslippage characteristics enables the TA-SIDI coating to demonstrate remarkable deicing capabilities (Fig. 6D). This approach simultaneously prevents ice accumulation through enhanced droplet removal and enables efficient ice detachment, offering an effective solution to ice accretion problems on wind turbine blades with strong potential for engineering implementation.

## Conclusion

We have developed a thermal responsive photothermal coating via a multiscale interpenetration of TCMs, robust skeleton, elastic network, and lubricant, which endows the coating with integrated performance of ultralow ice adhesion, excellent mechanical robustness, and thermochromic ability. This design successfully overcomes the critical challenge of photothermal coating with seasonal switching demands: Upon exceeding the critical temperature (*T*_c_ = 28 °C), the coating successfully limited the surface temperature to ~50 °C in summer compared to conventional photothermal surfaces (~90 °C), addressing the overheating challenge. In a freezing environment, 0.05 W/cm^2^ weak solar irradiation successfully raised the coating temperature to 7.6 °C and facilitated complete ice shedding from the rotating rotor within 418 s, addressing the weak-light inefficiency during winter. The coating retained excellent photothermal absorbance (>93%), thickness reduction (<32 μm), and ultraslippage properties (sliding angle <8.3°) after 60 h of UV aging, 200 Taber abrasions, demonstrating its durability for long-term service under natural conditions. This study is poised to illuminate new insights into all-season self-adaptive photothermal materials, pioneering a new pathway for designing intelligent anti-icing coatings for wind turbines and low-altitude rotorcrafts.

## Methods

### Materials and chemicals

All of the solvents and reagents were obtained from commercial sources and used as purchased without further purification. Epoxy resin precursor (E51), PEA, TEOS, and dibutyltin dilaurate were purchased from MACKLIN. α, ω-PDMS with 5,000 viscosity was purchased from Shenzhen Ji-Peng Silicon Fluoride Materials Co., Ltd. TCMs were purchased from Shenzhen Huancaibs Technology Co., Ltd.

### Fabrication of TA-IDI, IDI, and PHC

Based on the previous work of our research group, the optimal ratio of E51 and α, ω-PDMS has been determined. According to the mass contents of TCMs in IPN, TCMs were added to the mixture of E51 and α, ω-PDMS. After the mixture was fully stirred with mechanical stirring, PEA (mass ratio of 1:3 to E51) and TEOS (mass ratio of 1:10 to α, ω-PDMS) were added to the mixture drop by drop. Finally, the mixture was poured on the substrate, and the sample was heated at 80 °C for 12 h to obtain TA-IDI. The TCMs with same mass fraction carbon nanotubes were replaced to obtain the PHC coating. The IPN coating without TCMs is IDI.

### Fabrication of TA-SIDI

Silicone oil was sprayed on the as-prepared TA-IDI, which was then placed horizontally at ambient temperature for 12 h. The superfluous silicone oil on the surface was wiped off, and only the naturally diffused silicone oil was retained.

### Fabrication of PU

White polyurethane paint was directly sprayed onto the aluminum substrate to obtain a PU coating.

### Fabrication of accelerated soiling evaluation based on ASTM D7897-18 standard

The stain resistance of the coating was evaluated according to the ASTM D7897-18 standard. Four polluting agents (dust, humic acid, inorganic salt, and carbon black, which are similar to atmospheric dust, soot, salt, and POM) were prepared to simulate the natural pollution. The preparation process of each pollutant is described below.

1. Dust: Fe_2_O_3_ (0.3 ± 0.02 g) was mixed with 1.0 ± 0.05 g of montmorillonite and 1.0 ± 0.05 g of bentonite, and 1 l of distilled water was added to obtain a suspension with a concentration of 2.3 ± 0.1 g·l^−1^.

2. Soot: Carbon black (0.26 ± 0.01 g) was mixed with 1 l of distilled water to obtain a stable 0.26 ± 0.01·g l^−1^ soot suspension.

3. Salt: NaCl (0.3 ± 0.03 g), 0.3 ± 0.03 g of NaNO_3_, and 0.4 ± 0.03 g of CaSO_4_·2H_2_O were dissolved in 1 l of distilled water to obtain a salt solution with a concentration of 1.0 ± 0.1·g l^−1^.

4. POM: Humic acid (1.4 ± 0.05 g) was mixed with 1 l of distilled water to obtain a particulate organic solution with a concentration of 1.4 ± 0.05·g·l^−1^.

### Characterizations

Morphologies were characterized by a focused ion beam scanning electron microscope (Helios G4CX). FT-IR spectroscopy was measured by a Thermo Scientific Nicolet iS20 with an attenuated total reflection model. Differential scanning calorimetry was measured by a NETZSCH STA 449 C. Contact angle and sliding angle were measured using a 6-μl deionized water droplet by contour image analysis (Software ImageJ 1.52n). The solar thermal experiments were conducted with a solar simulator (CEL-S00, CEAULIGHT, Beijing, China). Deicing properties were investigated using a custom-made low-temperature testing device. Ice adhesion force was measured by force gauges (SH-20N and SH-200N, Nscing Es Instrument Co., Ltd.). The ice detachment behaviors were measured on a rotor captured by a high-speed camera (Photron Nova S9). The coating thickness was measured by a film thickness tester (LS225, Shenzhen Linshang Technology Co., Ltd.).

## Data Availability

The data that support the findings of this study are available from the corresponding authors upon reasonable request.

## References

[1] Kreder MJ, Alvarenga J, Kim P, Aizenberg J. Design of anti-icing surfaces: Smooth, textured or slippery? Nat Rev Mater‌. 2016;1(1):15003.

[2] Lambley H, Graeber G, Vogt R, Gaugler LC, Baumann E, Schutzius TM, Poulikakos D. Freezing-induced wetting transitions on superhydrophobic surfaces. Nat Phys. 2023;19(5):649–655. 37205127 10.1038/s41567-023-01946-3 PMC10185467

[3] Li J, Ueda E, Paulssen D, Levkin PA. Slippery lubricant-infused surfaces: Properties and emerging applications. Adv Funct Mater. 2019;29(4):1802317.

[4] Dhyani A, Choi W, Golovin K, Tuteja A. Surface design strategies for mitigating ice and snow accretion. Matter. 2022;5:1423–1454.

[5] Wang T, Zheng Y, Raji A-RO, Li Y, Sikkema WK, Tour JM. Passive anti-icing and active deicing films. ACS Appl Mater Interfaces. 2016;8(22):14169–14173. 27192099 10.1021/acsami.6b03060

[6] Wang T, Feng H, Cao L, Zhao Z, Li W, Chen S. Mechanism and design strategy of ice-phobic surface: A comprehensive review. Adv Colloid Interface Sci. 2025;341:103478. 40139068 10.1016/j.cis.2025.103478

[7] Tian S, Li R, Liu X, Wang J, Yu J, Xu S, Tian Y, Yang J, Zhang L. Inhibition of defect-induced ice nucleation, propagation, and adhesion by bioinspired self-healing anti-icing coatings. Research. 2023;6:0140. 37214197 10.34133/research.0140 PMC10194051

[8] Wang L, Yin K, Li X, Huang Y, Xiao J, Pei J, Song X, Duan J-A, Arnusch CJ. Femtosecond laser ultrafast atomic scale renovating laser-induced graphene. Adv Funct Mater. 2025;35(43):2506215.

[9] Xie J, Wu H, Tao J, Lu Z, Liu X, Tong Y, Li S, Qi S, Ran Q. Meniscus-inspired segmented network intercalation strategy: Stiffened yet toughened toward healable antifouling materials. Adv Funct Mater. 2025;35(39):2503647.

[10] Wang L, Li D, Jiang G, Hu X, Peng R, Song Z, Zhang H, Fan P, Zhong M. Dual-energy-barrier stable superhydrophobic structures for long icing delay. ACS Nano. 2024;18(19):12489–12502. 38698739 10.1021/acsnano.4c02051

[11] Xuan S, Yin H, Li G, Zhang Z, Jiao Y, Liao Z, Li J, Liu S, Wang Y, Tang C, et al. *Trifolium repens* L.-like periodic micronano structured superhydrophobic surface with ultralow ice adhesion for efficient anti-icing/deicing. ACS Nano. 2023;17(21):21749–21760. 37843015 10.1021/acsnano.3c07385

[12] Xiao J, Yin K, Wang L, Pei J, Song X, Huang Y, He J, Duan J-A. Femtosecond laser atomic–nano–micro fabrication of biomimetic perovskite quantum dots films toward durable multicolor display. ACS Nano. 2025;19(25):23431–23441. 40536059 10.1021/acsnano.5c06945

[13] Tao J, Wu H, Chen S, Xie J, Liu X, Tong Y, Ran Q. High-durable and photothermal self-healing properties of a silicon-polyaniline superhydrophobic composite network inspired by worms. Nano Mater Sci. 2024; 10.1016/j.nanoms.2024.12.001.

[14] Chen T-L, Lin Y-P, Chien C-H, Chen Y-C, Yang Y-J, Wang W-L, Chien L-F, Hsueh H-Y. Fabrication of frog-skin-inspired slippery antibiofouling coatings through degradable block copolymer wrinkling. Adv Funct Mater. 2021;31(42):2104173.

[15] Zhang Z, Wei D, Zhang W, Shi Y, Wang Y, Lu T, Oliveira Henriques Moita AS, Wang G, Liu Y. Scalable robust and photothermal-responsive superhydrophobic coating with switchable wettability and dewetting ability for efficient anti-/deicing. Adv Funct Mater. 2025;36(32): Article e18852.

[16] Zhang P, He S, Zhang L, Wu J, Guo Z. Durable bionic honeycomb slippery liquid-infused porous surfaces with anti-icing and water-collecting properties. Chem Eng J. 2024;490: Article 151478.

[17] Wang D, Guo Z, Liu W. Bioinspired edible lubricant-infused surface with liquid residue reduction properties. Research. 2019;2019: Article 1649427. 31922129 10.34133/2019/1649427 PMC6946289

[18] Pei J, Yin K, Yao W, Fu Y, Yang P, Wang L, Huang Y, Liu X. Anomalous spontaneous entropy reduction phenomenon in oil droplet diffusion. Appl Phys Lett. 2025;127(18): Article 181603.

[19] Wang Z, Zhao Z, Wen G, Zhu Y, Chen J, Jing X, Sun S, Zhang L, Liu X, Chen H. Fracture-promoted ultraslippery ice detachment interface for long-lasting anti-icing. ACS Nano. 2023;17(14):13724–13733. 37403892 10.1021/acsnano.3c03023

[20] Wu H, Lu Z, Jin M, Xie J, Tao J, Yue C, Dong L, Ran Q. Nature-inspired dynamic nanoconfinement enables life-like mechanical adaptability and robust environmental resilience in polyurethane-urea elastomers. J Mater Chem A. 2025;13:39243–39253.

[21] Liu L, Chen S, Hu Y, Pan W, Dong T, Chen Y, Lin L, Wang L. Anti-/deicing membranes with damage detection and fast healing. Adv Funct Mater. 2024;34(40):2404760.

[22] Wang L, Yin K, Xiao J, Song X, Pei J, He J, Duan J-A. Femtosecond laser synthesis of multiscale high-entropy alloys/graphene composites for high-performance joule heating. Nat Commun. 2026;17(1):2121. 41776159 10.1038/s41467-026-70162-3 PMC12957300

[23] Zhao X, Yao C, Liu T, Hamill JC Jr, Ngongang Ndjawa GO, Cheng G, Yao N, Meng H, Loo Y-L. Perovskite solar cells: Extending the photovoltaic response of perovskite solar cells into the near-infrared with a narrow-bandgap organic semiconductor. Adv Mater. 2019;31(49):1970349. 10.1002/adma.201904494 31523862

[24] Cheng Y, Ma J, Luo H, Cai M, Xue T, Yu G, Ren Z, Song Y, Peng S, Zhang Y. Unraveling segregation behavior of inactive secondary phase driven by ion-competition reaction for perovskite-2D PbI_2_ heterojunction solar cells. Nano Energy. 2023;115: Article 108690.

[25] Niu W, Chen GY, Xu H, Liu X, Sun J. Highly transparent and self-healable solar thermal anti-/deicing surfaces: When ultrathin MXene multilayers marry a solid slippery self-cleaning coating. Adv Mater. 2022;34(10):2108232. 10.1002/adma.202108232 34963016

[26] Wu Y, Dong L, Shu X, Yang Y, Feng P, Ran Q. Recent advancements in photothermal anti-icing/deicing materials. Chem Eng J. 2023;469: Article 143924.

[27] Tao J, Wu H, Xie J, Lu Z, Li S, Jin M, Zhao H, Dong L, Chen S, Yang Y, et al. Mechanically interlocked bioinspired armor: Sea urchin-mimetic superhydrophobic coatings with ultrahigh durability for anti-icing/deicing. Small. 2025;21(35):2505827. 10.1002/smll.202505827 40641324

[28] Wu C, Geng H, Tan S, Lv J, Wang H, He Z, Wang J. Highly efficient solar anti-icing/deicing via a hierarchical structured surface. Mater Horiz. 2020;7:2097–2104.

[29] Ren Z, Niu S, Lv A, Liu X, Mu W, Liu T, Wang Q. Bioinspired photothermal superhydrophobic metamaterial with structured micro-nano crystal arrays for anti-/de-icing. Adv Mater. 2025;38(6): Article e16655. 41190866 10.1002/adma.202516655

[30] Li N, Zhang Y, Zhi H, Tang J, Shao Y, Yang L, Sun T, Liu H, Xue G. Micro/nano-cactus structured aluminium with superhydrophobicity and plasmon-enhanced photothermal trap for icephobicity. Chem Eng J. 2022;429: Article 132183.

[31] Liu J, Pei K, Zhou Y, Fu S, Ai S, Wang Y, Jiang H, Zhou Z, Guo Z. Bioinspired ultrasmall-bandgap MOF-integrated superhydrophobic textiles via in situ self-assembly: Enabling next-generation multifunctional smart textiles. Adv Funct Mater. 2025;36(11): Article e13624.

[32] Mao M, Wei J, Li B, Li L, Huang X, Zhang J. Scalable robust photothermal superhydrophobic coatings for efficient anti-icing and de-icing in simulated/real environments. Nat Commun. 2024;15(1):9610. 39505855 10.1038/s41467-024-54058-8 PMC11541590

[33] Hou M, Jiang Z, Sun W, Chen Z, Chu F, Lai N-C. Efficient photothermal anti-/deicing enabled by 3D Cu_2-x_ S encapsulated phase change materials mixed superhydrophobic coatings. Adv Mater. 2024;36(3):2310312. 10.1002/adma.202310312 37991469

[34] Zhou P, Jiang G, Wang Y, Tian Y, Zhang X. Self-adaptive and large-area sprayable thermal management coatings for energy saving. Nat Commun. 2025;16(1):3791. 40263304 10.1038/s41467-025-59259-3 PMC12015516

[35] Zhu K, Yao H, Song J, Liao Q, He S, Guang T, Wang H, Hao X, Lu B, Lin T, et al. Temperature-adaptive dual-modal photonic textiles for thermal management. Sci Adv. 2024;10(41):eadr2062. 39383222 10.1126/sciadv.adr2062 PMC11463281

[36] Perera DY. Physical ageing of organic coatings. Prog Org Coat. 2003;47(1):61–76.

[37] Zhang M, Sun B, Gu B. Accelerated thermal ageing of epoxy resin and 3-D carbon fiber/epoxy braided composites. Compos Part A Appl Sci Manuf. 2016;85:163–171.

[38] Liu P, Meng F, Barlow CY. Wind turbine blade end-of-life options: An economic comparison. Resour Conserv Recycl‌. 2022;180: Article 106202.

[39] Tao P, Ni G, Song C, Shang W, Wu J, Zhu J, Chen G, Deng T. Solar-driven interfacial evaporation. Nat Energy. 2018;3(12):1031–1041.

[40] Verma S, Das S, Mohanty S, Nayak SK. A facile preparation of epoxy-polydimethylsiloxane (EP-PDMS) polymer coatings for marine applications. J Mater Res. 2019;34(16):2881–2894.

[41] Hernández CS, Hernández MS, Cerritos RC, Elorza E, Mendoza-Miranda JM, Navarro R. DBTL as neutral catalyst on TEOS/PDMS anticorrosive coating. J Sol Gel Sci Technol. 2017;81:405–412.

[42] Jayakumar N, Karattu Veedu K, Gopalan NK. Durable hydrophobic coating based on cerium phosphate nanorod-siliconized epoxy for corrosion protection. ACS Appl Nano Mater. 2019;2(5):2689–2696.

[43] Lee J, Kim B, Lee JW, Hong CY, Kim GH, Lee SJ. Bioinspired fatty acid amide-based slippery oleogels for shear-stable lubrication. Adv Sci. 2022;9(8):2105528. 10.1002/advs.202105528 PMC8922109 35072365

[44] Zhang Z, Liu B, Fang C, Chen W, Gu Y, Li J. Study on high temperature resistance of composite materials in wind blades under desert environment. Compos Mater Sci Eng. 2024;103–107.10.19936/j.cnki.2096-8000.20240328.015

[45] Peng C, Chen Z, Tiwari MK. All-organic superhydrophobic coatings with mechanochemical robustness and liquid impalement resistance. Nat Mater. 2018;17(4):355–360. 29581573 10.1038/s41563-018-0044-2

[46] Liu B-Y, Wu J, Xue C-H, Zeng Y, Liang J, Zhang S, Liu M, Ma C-Q, Wang Z, Tao G. Bioinspired superhydrophobic all-in-one coating for adaptive thermoregulation. Adv Mater. 2024;36(31):2400745. 10.1002/adma.202400745 38810961

[47] Zhao X, Li T, Xie H, Liu H, Wang L, Qu Y, Li SC, Liu S, Brozena AH, Yu Z, et al. A solution-processed radiative cooling glass. Science. 2023;382(6671):684–691. 37943922 10.1126/science.adi2224

[48] Golovin K, Dhyani A, Thouless M, Tuteja A. Low–interfacial toughness materials for effective large-scale deicing. Science. 2019;364(6438):371–375. 31023920 10.1126/science.aav1266

[49] Meuler AJ, Smith JD, Varanasi KK, Mabry JM, McKinley GH, Cohen RE. Relationships between water wettability and ice adhesion. ACS Appl Mater Interfaces. 2010;2(11):3100–3110. 20949900 10.1021/am1006035

[50] Golovin K, Kobaku SPR, Lee DH, DiLoreto ET, Mabry JM, Tuteja A. Designing durable icephobic surfaces. Sci Adv. 2016;2(3):2375–2548. 10.1126/sciadv.1501496 PMC4795665 26998520

[51] Shi J, Fulford M, Li H, Marzook M, Reisjalali M, Salvalaglio M, Molteni C. Investigating the quasi-liquid layer on ice surfaces: A comparison of order parameters. Phys Chem Chem Phys. 2022;24(20):12476–12487. 35576067 10.1039/d2cp00752e

[52] Asakawa H, Sazaki G, Nagashima K, Nakatsubo S, Furukawa Y. Two types of quasi-liquid layers on ice crystals are formed kinetically. Proc Natl Acad Sci USA. 2016;113(7):1749–1753. 26831089 10.1073/pnas.1521607113 PMC4763737

[53] Zhao Z, Chen H, Liu X, Liu H, Zhang D. Development of high-efficient synthetic electric heating coating for anti-icing/de-icing. Surf Coat Technol. 2018;349:340–346.

[54] Schubel PJ, Crossley RJ. Wind turbine blade design review. Wind Eng. 2012;36(4):365–388.

