# Supplementary material for: Robust Thermochromic Photothermal Coating with Ultraslippery Anti-icing/Deicing and All-Season Temperature Regulation Performance
Source: Research (Wash D C). 2026 Jun 1;9:1285. doi: 10.34133/research.1285 (PMC13223374; doi:10.34133/research.1285)
Supplement: Supplementary 1 — Figs. S1 to S18 Movies S1 and S2 [file research.1285.f1.zip › Supplemental Materials.pdf]

Supplementary Materials

Robust thermochromic photothermal coating with ultraslippery anti-icing/de-icing and all-season temperature regulation performance

Shize Sun[‡, 1], Xiaolin Liu[‡, *, 1], Zelinlan Wang[1], Changjun Yang[1], Jichen Chen[1,2], Zehui Zhao[3], Junbo Liu[1], Liwen Zhang[1], Huawei Chen[*, 1]

1 Institute of Bionic Micro-Nano Systems, School of Mechanical Engineering and Automation, Beihang University, Beijing 100191, China

2 State Key Laboratory of Bioinspired interfacial Materials Science, Bioinspired Science Innovation Center, Hangzhou International innovation Institute, Beihang University, Hangzhou 311115, China

3 College of Mechanical and Transportation Engineering, China University of Petroleum, Beijing 102249, China

‡ These authors contribute equally to this work.

*Corresponding authors: Chenhw75@buaa.edu.cn, Liuxl7@buaa.edu.cn,

Supporting Figures

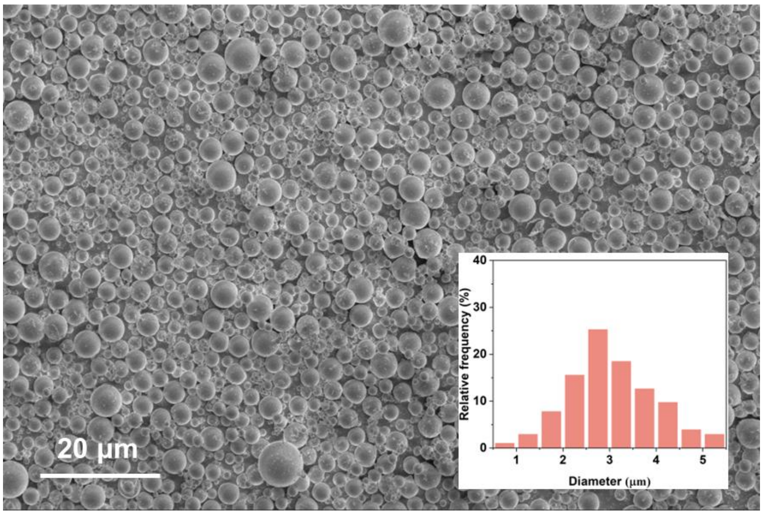

Figure S1. The SEM image of TCMs shows the microcapsules' size concentrated at 3 μm.

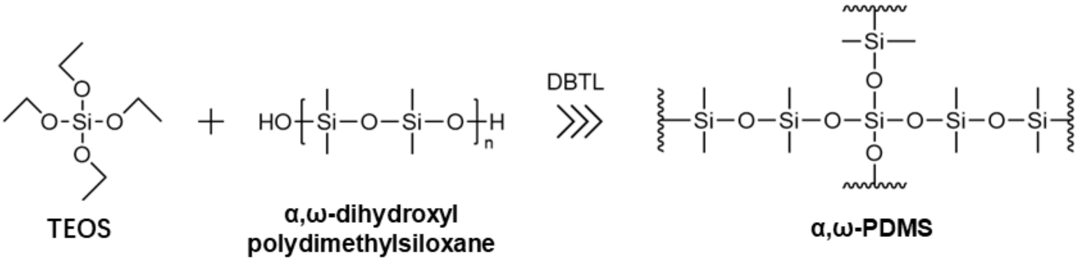

Figure S2. Chemical reaction formula between TEOS and α, ω-dihydroxyl polydimethylsiloxane to generate α, ω-PDMS.

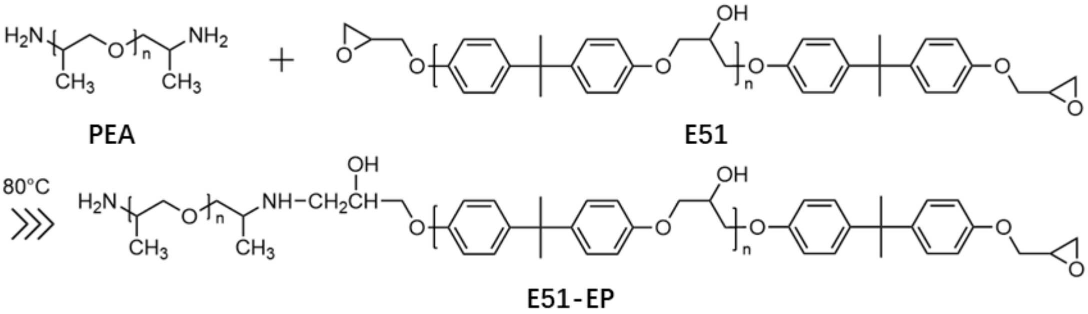

Figure S3. The curing Chemical reaction formula of E51 and PEA.

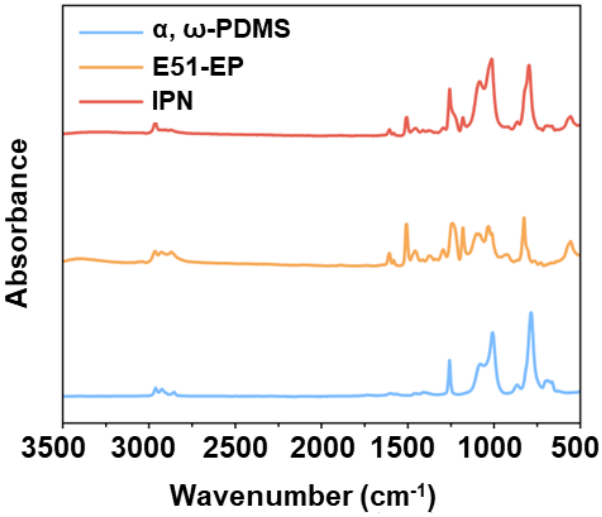

Figure S4. FT-IR spectrum of IPN including E51-EP, α, ω-PDMS, and IPN.

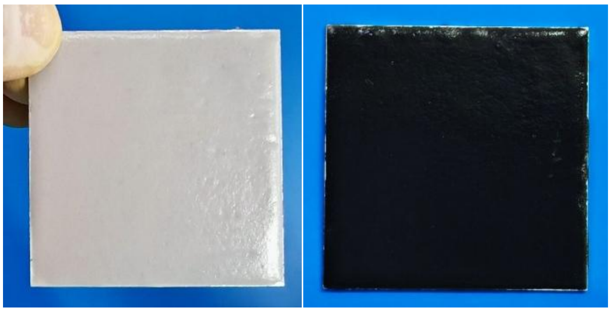

Figure S5. Color response of the TA-SIDI coating at different temperatures. (Left: T > T_c; Right: T < T_c).

Ambient temperature is critical to the reliability of composite wind turbine blades. According to GB/T 25384-2018 and IEC 61400-23:2014 standards, 20–25 °C represents the optimal operating range for a composite blade, delivering the best mechanical performance, longest fatigue life, and most stable bonding system. This is further supported by the wind farm failure analysis, which indicates the lowest failure rate at 20–25 °C. As illustrated in Figure 3e, the TA-SIDI coating can elevate the surface temperature to 28 °C within 600 s at −10 °C to ensure effective anti-icing performance, and switch to anti-overheating mode above 28 °C. TA-SIDI exhibits a faded color and reduced photothermal efficiency at 25 °C, the optimal service temperature (Figure 2d). To maintain efficient heating while keeping the internal temperature near 25 °C, the transition temperature was set slightly higher. Therefore, 28 °C is chosen as the thermochromic transition temperature to keep the blade operating within the optimal

20–25 °C range and improve its operational reliability.

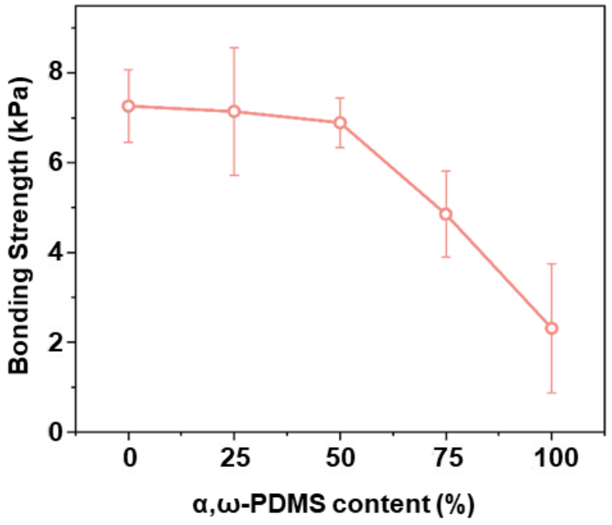

Figure S6. Bonding strength of Pure E51-EP.

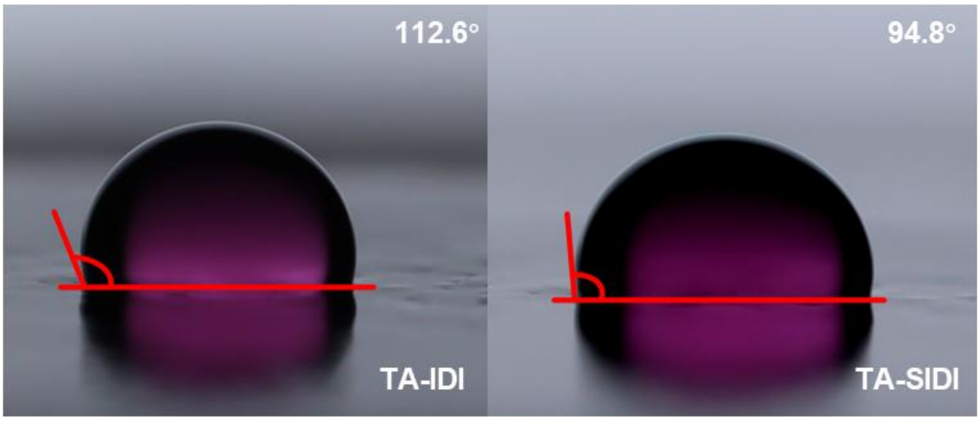

Figure S7. Contact angle of TA-IDI and TA-SIDI.

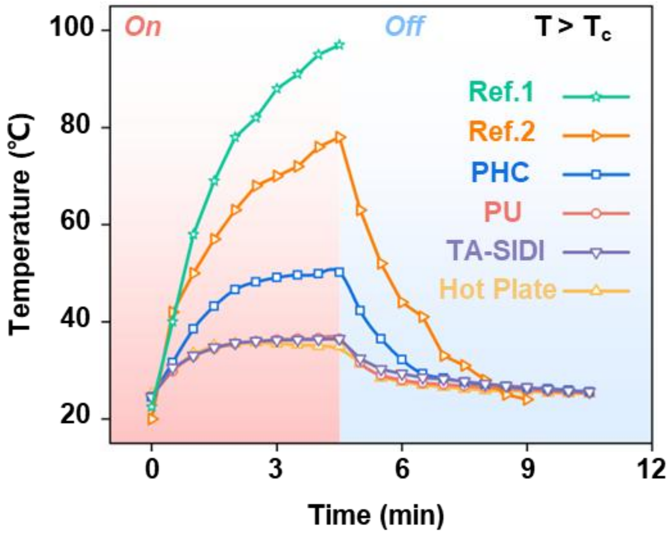

Figure S8. Comparison of the temperature variation of TA-SIDI with previously reported high efficient photothermal strategies at 0.10 W /cm^2 solar intensity (TA-SIDI(RT): TA-SIDI under 23 °C room temperature; TA-SIDI(HT): TA-SIDI under 40 °C high temperature)[15, 29, 31].

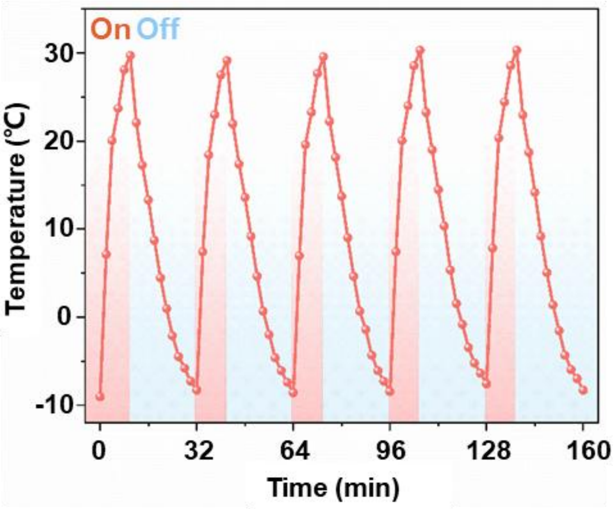

Figure S9. The temperature variation of TA-SIDI during the cyclic heating-cooling process at 0.20 W /cm^2 solar intensity.

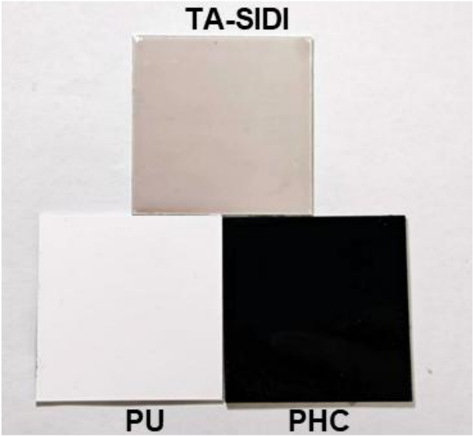

Figure S10. The digital photos of TA-SIDI, PHC, and PU in the open air.

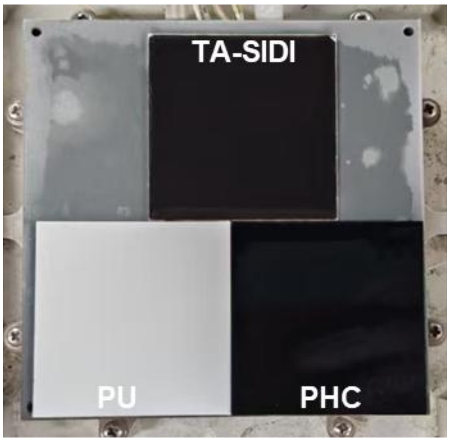

Figure S11. The digital photos of TA-SIDI, PHC, and PU on the cold plate.

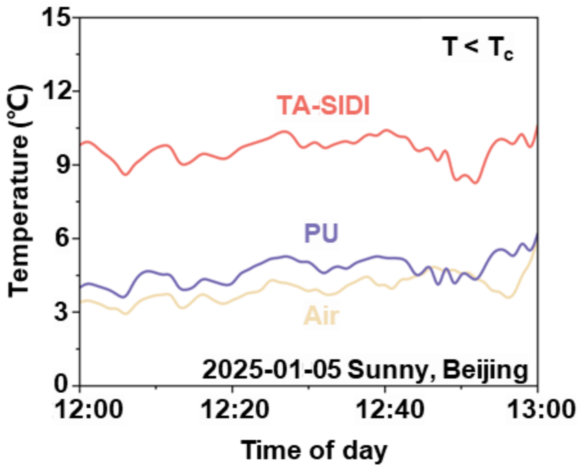

Figure S12. The real-time temperature record of the TA-SIDI, PU, and air (Beijing, 2025-01-05, Sunny).

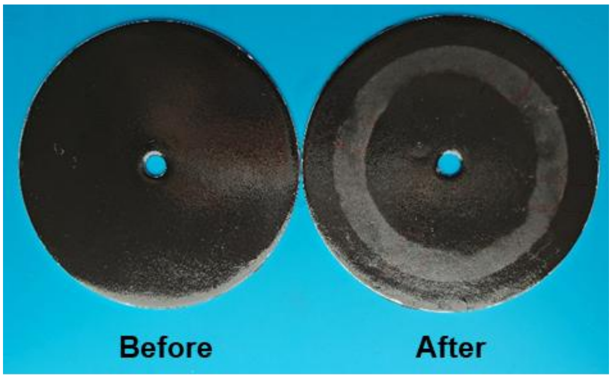

Figure S13. The digital photos of TA-SIDI, TA-IDI, before and after the Taber test.

Abrasion resistance is a critical parameter for evaluating the suitability of coatings for practical applications. In this study, the abrasion resistance of the coating was evaluated using a Taber abrasion tester per established methods[45]. The coating was applied to circular aluminum or brass substrates with an outer diameter of 108 mm and an inner diameter of 8 mm. Abrasion tests were conducted using CS-17 grinding wheels under a load of 250 g per wheel. Following the ASTM standard, one full rotation of the substrate was considered one cycle, and the tests were performed at a rotational speed of 99 cycles per minute. The interfacial properties, including ice adhesion strength (τ_{ice}), surface roughness (SA), and coating thickness, were periodically measured in the abraded regions. It should be noted that the grinding wheels were cleaned and dried thoroughly after each test to ensure consistent performance.

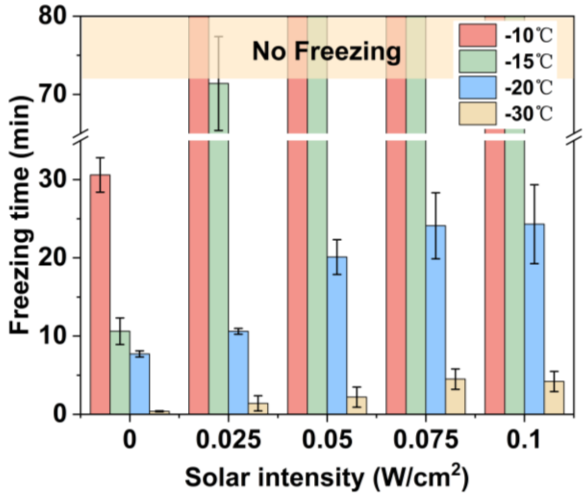

Figure S14. The freezing time of TA-SIDI with different solar intensities under -10, -15, -20, and -30°C.

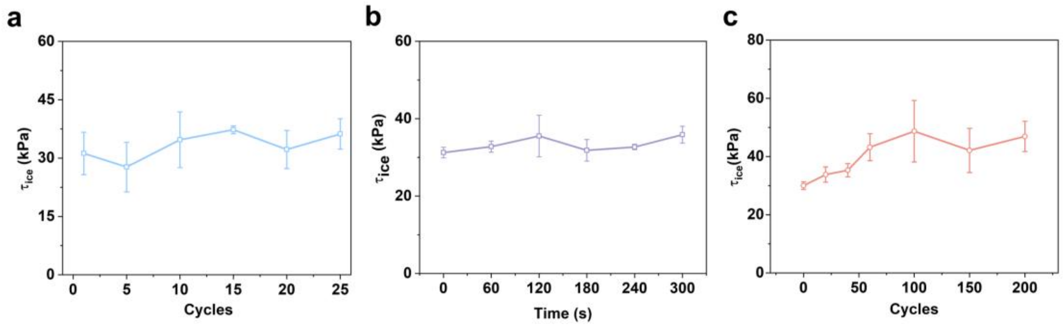

Figure S15. (a) τ_{ice} of TA-SIDI after 25 de-icing cycles. (b) τ_{ice} of TA-SIDI after 300s water impact. (c) τ_{ice} on the abrasion areas of TA-SIDI after 200 abrasion cycles.

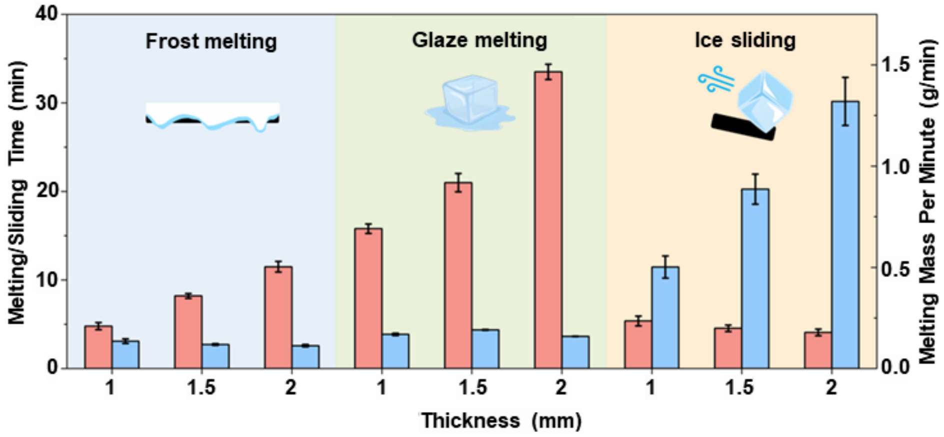

Figure S16. Frost melting, Glaze melting, and Ice sliding time of TA-SIDI with different ice thickness.

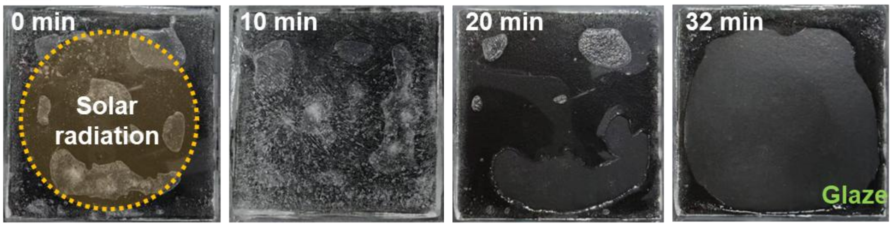

Figure S17. Glaze melting performance along with time during the deicing process.

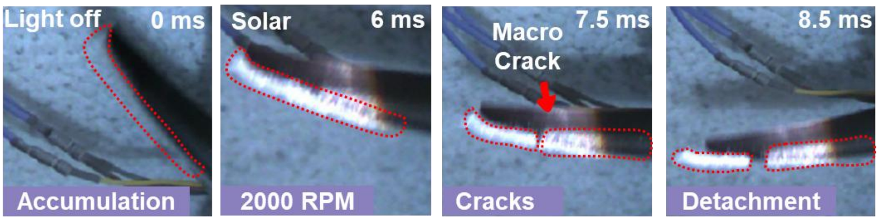

Figure S18. Ice detachment behavior on a blade with TA-SIDI coating at 1000 rpm under solar irradiation.

Supporting Movies

Movie S1: Color change process of TA-SIDI between low and high temperature environments. (MP4)

Movie S2: Experimental results of the detachment process of ice (MP4)
